# Sub-microscopic *Plasmodium falciparum* parasitaemia, dihydropteroate synthase (dhps) resistance mutations to sulfadoxine–pyrimethamine, transmission intensity and risk of malaria infection in pregnancy in Mount Cameroon Region

**DOI:** 10.1186/s12936-023-04485-7

**Published:** 2023-03-02

**Authors:** Harry F. Mbacham, Diange M Mosume, Tobias O. Apinjoh, Vincent N. Ntui, Marcel N. Moyeh, Laken N. Kalaji, Godlove B. Wepnje, Stephen M Ghogomu, Jodie A Dionne, Alan T.N. Tita, Eric A. Achidi, Judith K. Anchang-Kimbi

**Affiliations:** 1 Department of Animal Biology and Conservation, University of Buea, Buea, Cameroon; 2 Department of Biochemistry and Molecular Biology, University of Buea, Buea, Cameroon; 3 Department of Medicine, University of Alabama at Birmingham, Birmingham, USA; 4 Department of Obstetrics and Gynecology, University of Alabama at Birmingham, Birmingham, USA

**Keywords:** Pregnancy, IPTp-SP, *P. falciparum*, Sub-microscopic parasitaemia, *Dhps* resistant mutations, Cameroon

## Abstract

**Background:**

*Plasmodium falciparum* resistance to intermittent preventive treatment with sulfadoxine-pyrimethamine (IPTp-SP) continues to spread throughout sub-Saharan Africa. This study assessed the occurrence of microscopic and sub-microscopic *P. falciparum* parasitaemia, dihydropteroate synthase mutations associated with resistance to SP and maternal anaemia in the Mount Cameroon area.

**Methods:**

Consenting pregnant women living in semi-rural and semi-urban/urbanized settings were enrolled in this cross-sectional study. Socio-demographic, antenatal and clinical data were documented. Microscopic and sub-microscopic parasitaemia were diagnosed using peripheral blood microscopy and nested polymerase chain reaction (PCR) respectively. The *dhps* mutations were genotyped by restriction fragment length polymorphism analysis. The presence of A437G, K540E, and A581G was considered a marker for high-level resistance. Haemoglobin levels and anaemia status were determined.

**Results:**

Among the women, the prevalence of microscopic and sub-microscopic *P. falciparum* infection were 7.7% (67/874) and 18.6% (93/500) respectively. Predictors of microscopic infection were younger age (< 21 years) (AOR = 2.89; 95% CI 1.29–6.46) and semi-rural settings (AOR = 2.27; 95% CI 1.31–3.96). Determinants of sub-microscopic infection were the rainy season (AOR, 3.01; 95% CI 1.77–5.13), primigravidity (AOR = 0.45; 95% CI 0.21–0.94) and regular ITN usage (AOR = 0.49; 95% CI 0.27–0.90). Of the145 *P. falciparum* isolates genotyped, 66.9% (97) carried mutations associated with resistance to SP; 33.8% (49), 0%, 52.4% (76) and 19.3% (28) for A437G, K540E, A581G and A437G + A581G respectively. The A581G mutation was associated with ≥ 3 SP doses evident only among sub-microscopic parasitaemia (P = 0.027) and multigravidae (P = 0.009). Women with microscopic infection were more likely from semi-rural settings (AOR = 7.09; 95% CI 2.59–19.42), to report history of fever (AOR = 2.6; 95% CI 1.07–6.31), to harbour parasites with double resistant mutations (AOR = 6.65; 95% CI 1.85–23.96) and were less likely to have received 2 SP doses (AOR = 0.29; 95% CI 1.07–6.31). Microscopic infection decreased Hb levels more than sub-microscopic infection.

**Conclusion:**

The occurrence of sub-microscopic *P. falciparum* parasites resistant to SP and intense malaria transmission poses persistent risk of malaria infection during pregnancy in the area. ITN usage and monitoring spread of resistance are critical.

**Supplementary Information:**

The online version contains supplementary material available at 10.1186/s12936-023-04485-7.

## Background

In areas of sustained malaria transmission, pregnant women and their neonates are predisposed to high risk of adverse outcomes such as malaria, placental infection, anaemia, preterm delivery, and neonatal low birth weight (LBW) and mortality, respectively [1]. The global number of pregnancies at risk of *Plasmodium falciparum* and *Plasmodium vivax* malaria decreased between 2000 and 2020, but exceptionally, the number of pregnancies at risk of moderate to high *P. falciparum* transmission in sub-Saharan Africa increased from 37.3 M to 52.4 M in 2020. Arguably, interventions that reduce the risk of malaria in pregnancy are more important today as ever [1]. Protection from these pregnancy associated malaria (PAM)-attributable adverse outcomes relies on the use of long-lasting insecticidal nets (LLINs), IPTp-SP, and effective case management through prompt diagnosis and treatment [2]. The World Health Organization (WHO) recommends at least three doses of SP to all pregnant women during antenatal care (ANC) clinic visits starting early second trimester, till the time of delivery, provided each dose is given at least a month apart [3]. In 2022, the WHO reiterated that, in malaria-endemic areas, pregnant women of all gravidities should be given antimalarial medicine at predetermined intervals to reduce disease burden in pregnancy and adverse pregnancy and birth outcomes [4].

The rising burden of *P. falciparum* resistance to essential anti-malarial drugs is a major challenge to the global fight against malaria [5]. Parasite resistance to IPTp-SP is driven by increasing prevalence of high-level resistant parasite strains, mostly in eastern and southern Africa [6, 7]. The selection of these resistant parasite strains can be alarming rapid and thus continues to threaten effectiveness of SP throughout sub-Saharan Africa [8, 9]. Alternative drug regimens have been evaluated for IPTp. Dihydroartemisinin-piperaquine (DP) is the most promising candidate, given that it is more effective in preventing malaria infection than is SP [10, 11]. Nevertheless, compared with IPTp-DP, IPTp-SP appears to have potent non-malarial than anti-malarial effects on birthweight [12] and may explain why IPTp-SP remains protective against low birthweight risk in areas with high parasite resistance to SP [6, 13, 14].

As several studies continue to report the effectiveness of IPTp with SP, validation of its usefulness in varying dynamics of PAM is necessary [15]. Malaria infection commonly occurs during pregnancy, but routine microscopic and rapid diagnostic tests fail to detect most episodes. Molecular detection of MiP has a much higher sensitivity and is revealing a widespread occurrence of submicroscopic infections below the detection threshold of microscopy [16–19]. This hidden infection can persist as chronic infection with ensuing negative clinical impact on pregnancy. The deleterious effect of submicroscopic infections (either in peripheral blood or placenta) on maternal anaemia and LBW is increasingly reported [17–22]. Moreover, hidden human infectious reservoirs could potentially contribute to the persistence of transmission as well result in drug resistance emergence and/or spread [23]. Abdul-Ghani and colleagues [24] suggested possible build-up of resistance to anti-malarial drugs among submicroscopic parasite populations and the emergence of submicroscopic resistant parasites as an important factor limiting the effectiveness of malaria elimination strategies.

The WHO recommends member states to closely monitor the efficacy of essential anti-malarial drugs and use resistance levels to inform policymaking at the country-level [3]. The prevalence and frequency of molecular markers are evaluated to track and measure resistance levels of drug-resistant *P. falciparum* [7, 25]. Parasite susceptibility to SP is influenced by mutations in two genes, for two enzymes of the folate pathway, dihydropteroate synthase (DHPS) and dihydrofolate reductase (DHFR), which have been strongly associated with resistance to sulfadoxine and pyrimethamine respectively. Furthermore, the number and types of mutant codons increases the intensity of the resistance to SP [26]. The quintuple mutations (five mutations including three *dhfr* mutations (N51I, C59R, S108N) together with two *dhps* mutations (A437G, K540E) are associated with mid-level resistance, whereas sextuple mutations (six mutations including the quintuple mutations in addition to A581G) being linked with high-level resistance [7]. The association between these mutations and SP is complex as these mutations may cause a distortion on SP efficacy [13]. Studies in east and southern Africa have linked predominance of highly resistant strains carrying *pfdhps* K540E and A581G mutations to reduction in parasitological efficacy of IPTp-SP in these regions [13, 26–29]. Thus, the surveillance and reporting of K540E and A581G mutations has a central role in IPTp-SP policy decisions [3, 25]. Of equal importance is the detection and monitoring of reservoir sub-microscopic parasites in the context of the effectiveness of IPTp-SP. A combined assessment of molecular markers of resistance in sub-microscopic and microscopic malaria infections could enhance identification of the true burden of resistant parasites in endemic settings.

Meta-analysis studies have revealed a heterogenous pattern of *P. falciparum* resistance to SP across Africa [6, 7]. Meanwhile high-level resistance to SP has increased in eastern Africa, frequency of mid-level resistance is increasing in Central Africa [6, 7, 30–32] but remain largely unchanged in western Africa [7, 33]. There is still limited evidence on SP effectiveness for IPTp due to unavailability of data on high-level resistance in most locations of eastern Africa and central Africa [31, 32, 34, 35] where the largest proportion of *P. falciparum* infection prevails on the continent [36]. Cameroon is one of the central Africa countries where regional and national representative data on molecular markers of high resistance to IPTp-SP are limited [37, 38]. The Southwest Region of Cameroon is characterized by perennial malaria transmission which is intense in the rainy season, and prevalence of infection varies from meso-hyperendemicity in the mount Cameroon area [40, 41]. In the Mount Cameroon area, we observed beneficial association between the use of IPTp-SP (≥ 2 doses) and LBW considering that the risk of PM infection increased by 42% in women who received ≥ 3 doses of SP compared with those who had ≤ 2 doses [39]. Notably, these findings are consistent with extensive reports in areas with high parasite resistance to SP [6, 14]. Chico et al.[13] suggests the need for pregnancy studies to define specific 581G prevalence threshold where new strategies should be deployed. Accordingly, it is important to determine the 581G prevalence level at which IPTp-SP continues to protect against LBW in the study area.

To elucidate the protective effect of IPTp-SP in the mount Cameroon area, firstly, this study determined the prevalence of microscopic and sub-microscopic *P. falciparum* infection, and their determinants in pregnant women from semi-urban/urbanized and semi-rural settings as well as across the two seasons (rainy and dry). Secondly, the levels of *Pfdhps* A437G, K540E, and A581G mutations associated with resistance to SP were determined in isolates from infected women and their occurrence assessed in relation to varying doses of SP and malaria parasitaemic status.

## Methods

### Study area

**Fig. 1:**
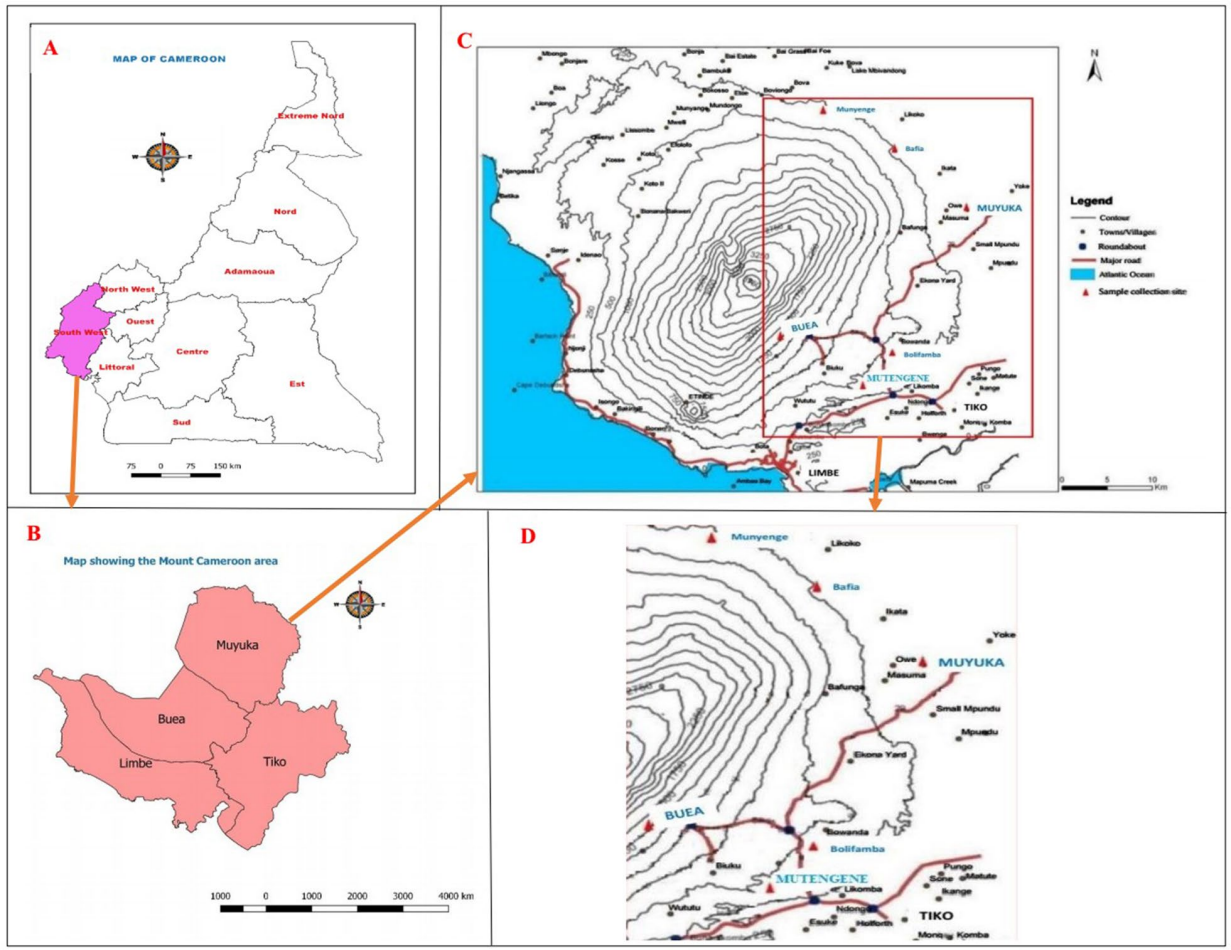
Map of Mount Cameroon area (Remote sensing unit-University of Buea, Cameroon)

Cameroon is in the central region of the African continent. The study was carried out in the Mount Cameroon area, Southwest Region. Pregnant women were enrolled from five medical facilities located across the eastern flank of Mount Cameroon which is characterized by varying levels of malaria transmission intensity [35, 36]: Muyuka District Hospital (MDH), Mutengene Medical Centre (MMC), Bolifamba Health Centre (BHC), Buea, Munyenge Integrated Health Centre (MIHC) and Buea road Integrated Health Centre (PMI) (Fig. 1). The localities of these facilities have been described elsewhere [39]. The Mount Cameroon region has an equatorial climate made up of a long rainy season that spans from March to October with maximum rainfall in August and September. The dry season starts in late October and ends in February. Malaria burden declined substantially from 2006 to 2019 [41, 42], mainly attributed to implementation of artemisinin-based combination therapy (ACT) and the wide scale distribution of long-lasting insecticidal nets. Placenta malaria infection has been used to characterize MiP in the Mount Cameroon area and findings reveal decreased trend from 2007 to 2017 (25.5–18.5%) [39, 43]. Nonetheless, infection prevalence remains higher in the semi-rural (22%) than in semi-urban (15%) settings [39, 40]. The scaled up of IPTp–SP uptake (≥ 3 doses) (47%) and ITN usage (67.7%) [39, 44, 45] has contributed to the overall reduction in infection prevalence in the region.

### Ethics statement

Ethical approval for this study was granted by the Institutional Review Board (IRB) of the University of Buea (Ref No: 2016/0351/UB/ FHS/IRB), meanwhile administrative authorization was obtained from the Southwest Regional Delegation of Public Health. Written informed concern was obtained from all the study participants before enrolment.

### Study population and design

Consenting pregnant women with a gestation age (GA) of at least 36 weeks attending ANC at the various health facilities were enrolled consecutively in a prospective cross-sectional survey from November 2016 to December 2017. Gestational age was calculated based on the last menstrual period or by the fundal height if the last menstrual period was not known. Pregnant women resident in semi-rural and semi-urban/urbanized settings were considered to avoid bias in analysis and interpretation of data. Enrolment of women with GA of at least 36 weeks ensured the opportunity to receive adequate SP doses [46]. Women with evidence of complicated pregnancy (hypertension, preeclampsia, diabetes) and HIV-infected women on cotrimoxazole were exempted from the study. Information on socio-demographic, obstetric/gynaecologic and number of SP doses intake were verified from individual ANC clinic record cards and documented, whereas participants were queried regarding ITN use the previous night to assess ITN usage. All women were examined for fever (defined as an axillary temperature ≥ 37.5 °C).

### Sample collection and laboratory analyses

At enrolment, about 2 mL of venous peripheral blood was collected for blood smear microscopy, preparation of dried blood spots (DBS) on 3MM Whatman filter paper (Whatman^®^ No. 3, (Sigma-Aldrich, Germany) for *P. falciparum* detection, genotyping by nested polymerase chain reaction (nPCR) and measurement of haemoglobin concentrations. Thick blood smear was prepared by placing a small drop of well mixed venous blood at the centre of a sterile labelled slide. Using the edge of another slide, the blood was spread in a circular pattern to a size of a coin (1.5 cm^2^). The smears were air-dried and stained with Giemsa (Sigma-Aldrich, St. Louis, MO). Dried blood spots were prepared by making three blood spots of the same participant on a well labelled 3MM Whatman filter paper and allowed to air-dry. The DBS on the filter paper were placed in sterile sealed plastic bags and stored at room temperature for further processing.

#### Microscopy

The stained thick blood smears were examined using a 100 × oil immersion objective to detect and quantify parasitaemia using an estimated white blood cell count of 8000 per microlitre as described previously [43]. A diagnosis of microscopic infection was made when asexual stage malaria parasites were detected on a thick smear as were a negative slide after examining 200 high power fields. Two microscopists read all slides independently; in cases of discordant readings, a third expert reader was referred.

#### Haematology

Haemoglobin levels were obtained using a HemoCue analyzer (HemoCue, Angelholm, Sweden) and maternal anaemia defined as haemoglobin level less than 11 g/dL [47].

#### Diagnosis of P. falciparum by nested-PCR

The DBS samples corresponding to positive and negative blood smear samples were analysed for *P. falciparum* parasitaemia. Genomic DNA was extracted from DBS samples by boiling in Chelex-100 ((Bio-Rad, Berkeley California,USA) as described elsewhere [48, 49]. DNA extracts were stored at − 20 °C until further use. *Plasmodium falciparum* was detected by nested PCR amplification (Bio-Rad T100™ thermal Cycler, Berkeley California, USA) of the 18 small sub-unit ribosomal RNA (18S rRNA) gene [48] using specific primers (Inqaba Biotec Pretoria, South Africa). Amplification conditions are shown in Additional file 1. Briefly, amplification was done at a total volume of 25μL which included 12.5μL of One Taq quick load 2X with standard buffer (New England Bio Labs), 0.5μL of the forward and reversed primers, 3μL of the DNA template and the rest filled with DNase free water. The amplification conditions for the primary and nested PCR were; Primary PCR of 25 cycles (initial denaturation-94 ºC for 3 min, denaturation-94 ºC for 30 s, annealing-55 ºC for 1 min, extention-68 ºC for 1 min, final extention-68 ºC for 3 min) and nested PCR of 30 cycles (initial denaturation-94 ºC for 3 min, denaturation-94 ºC for 30 s, annealing-61 ºC for 1 min, extention-68 ºC for 1 min, final extention-68 ºC for 3 min). The amplicon was separated in a 2% agarose gel electrophoresis alongside a 100 bp molecular weight maker and observed using a gel documentation system (Molecular Image® Gel Doc^™^ XR + System with Image Lab^™^ software, Bio-Rad, Berkeley California, USA). An amplified 205 bp indicated *P. falciparum* infection (Additional file 2(1)) The Nested PCR assay presented allows the detection and identification of *P. falciparum* at a lower limit of 1–10 parasites/μL [48].

### Amplification and restriction enzyme polymorphism of P. falciparum dhps resistant alleles

Single nucleotide polymorphism of the *pfdhps* gene fragments spanning codons 437–581 were genotyped by nested polymerase chain reaction followed by allele-specific restriction analysis (ASRA) using sequence specific primers obtained from Inqaba Biotec (Pretoria, South Africa). Restriction fragment length polymorphisms of PCR-generated amplicons identified mutations at gene regions covering codons 437, 540 and 581 as described by Mbugi et al. [50]. The primer pair R1 + R2 was used as the forward and reverse primers for the primary (nest I) PCR reaction to amplify the *dhps* domain. In the secondary (nest II) PCR reaction, the primer pair K1 + K2 (Additional file 1) was used for amplification of regions on the *dhps* domain associated with the 437 and 540 genes (indicated by an amplified 438 bp fragment). The primer pair L1 + L2 (Additional file 1) was used to amplify region of the *dhps* domain associated with the 581 gene (indicated by an amplified 161 bp fragment) [50].The primary and secondary PCR were performed at a volume of 25 μL which included 12.5 μL of One Taq quick load 2X with standard buffer (New England BioLabs, Inc.), 0.5μL of the forward and reversed primers, 3 μL of the DNA template and the rest filled with DNase free water. Specific-site restriction enzymes were used to digest secondary (nest II) PCR amplicons. The three restriction enzymes used were AvaII, FokI and BstUI to identify the A437G, K540E and A581G mutations, respectively. According to the manufacturer’s protocol, a total of 10μL of the PCR amplicon was incubate with specific restriction enzymes at specific temperatures in a final reaction volume of 20 μL (PCR amplicon: 10 μL, DNase water: 7.6 μL buffer: 2 μL enzyme: 0.4 μL). The digested products were separated in a 3% agarose gel electrophoresis alongside a 100 bp molecular weight maker and observed using a gel documentation system (Molecular Image^®^ Gel Doc^™^ XR + System with Image Lab^™^ software, Bio-Rad, Berkeley California, USA). Variation at codons 438 bp and 161 bp were indicative of mutations in the *dhps* genes studied (Additional file 2 (2a–c)) similar to previous studies [50–52].

### Outcome variables

The primary outcomes were microscopic and sub-microscopic *P. falciparum* parasitaemia and *Pfdhps* (437, 540 and 581) mutations. Secondary outcomes included maternal haemoglobin levels and anaemia.

#### Definition of variables

- 1. Microscopic infection (smear positive, confirmed by PCR) and sub-microscopic infection (smear negative, but PCR positive).
- 2. The prevalence of any resistant allele was defined as the proportion of *P. falciparum* isolates carrying any mutant parasite clone.
- 3. Fever was defined as a measured axillary temperature ≥ 37.5 °C.
- 4. Maternal anaemia was defined as haemoglobin < 11.0 g/dL.
- 5. ITN usage was considered as ITN use the previous night.
- 6. IPTp -SP uptake was defined as any dose of SP received by the time of study enrolment.

Independent variables considered in the study were age group (< 21, 21–25, > 25 years), seasonal status (rainy and dry season), study area setting (semi-rural, semi-urban/urbanized), gravidity status (primigravid, secundigravid and multigravid), marital status (married and single), ANC clinic visits (< 4 and ≥ 4 visits), IPTp-SP status (≤ 1 dose, 2 doses and ≥ 3 doses of SP).

### Data analysis

**Fig. 2:**
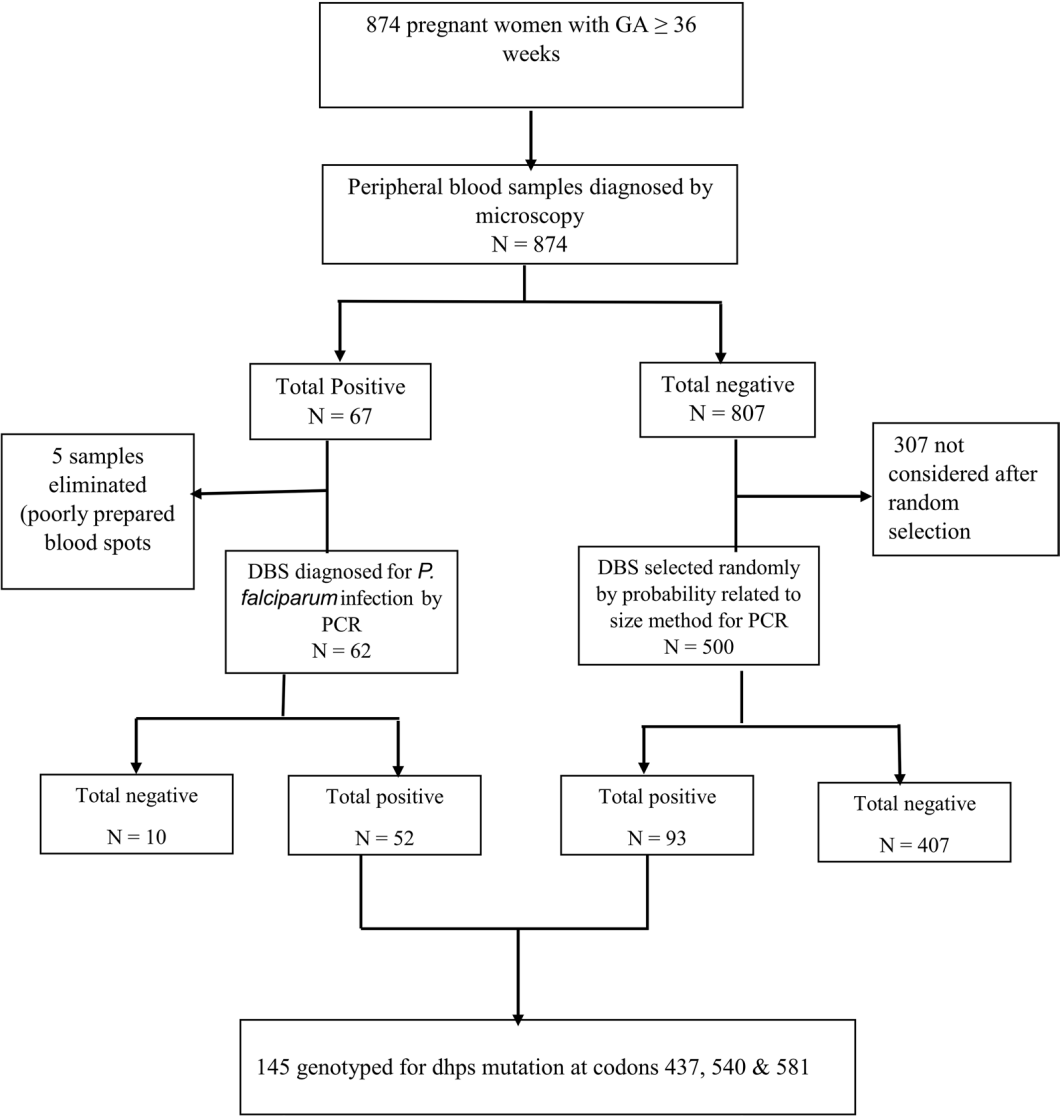
Flow diagram showing sample selection for the determination of *P. falciparum* microscopic and submicroscopic parasitaemia and genotyping of *Pfdhps* mutations in maternal peripheral blood

The analytical population consisted of 874 women diagnosed for microscopic infection, randomly selected 500 microscopic-negative women diagnosed for sub-microscopic infection and PCR-confirmed parasite isolates from 145 infected participants for detection of *dhps* mutations (Fig. 2). Data was entered, validated, and analysed using SPSS version 23 (IBM SPSS, Chicago, IL, USA). Sociodemographic, ANC and clinical characteristics were summarized using descriptive statistics. To determine the predictors of microscopic and submicroscopic malaria parasitaemia in pregnant women, covariates were fitted in the multivariable logistic regression analysis. Analysis of variance and Pearson chi-square analysis was used to assess the effect of microscopic and sub-microscopic infection on haemoglobin levels and anaemic status. The prevalence of any resistant allele was calculated, and proportions of each mutant allele assessed in relation to the different doses of SP using Pearson chi-square test. The distribution of malaria parasite infection in relation to age, setting, season, gravidity, IPTp-SP doses, ITN usage, fever history and resistant mutations was explored using logistic regression analysis. The risk of infection was measured as adjusted odds ratios (AOR). Statistical analysis was done at 95% confidence interval and statistical significance was set at p < 0.05.

## Results

Table 1 shows the distribution of baseline characteristics of pregnant women living in semi-rural and semi-urban/urbanized settings in the Mount Cameroon area. Mean maternal age was 25.98 ± 5.52 years (range 14–44). As expected, maternal socio-demographic characteristics differed between semi-rural and semi-urban/urbanized areas. Mean number of ANC visits per woman was 3.9 (range, 1–9), and 63.3% (553/874) of the women had made at least 4 complete visits during pregnancy. There was borderline significance (p = 0.049) in ANC attendance between the two settings. Among the women, 93.4% (816/874)   had taken at least one dose of SP (range: 1–5 doses) and about 74.9% (655/874)   received two or more SP doses by 36 weeks of gestation. Uptake ≤ 1 SP was common in the semi-rural setting. About 60% (522/874) of women stated using an ITN the preceding night with less usage among semi-urban residents. Semi-rural women experienced more febrile illness, fever history and anaemia than semi urban dwellers (Additional file 3) similar to previous reports [39].

**Table 1:** General characteristics of the enrolled (N = 874) and infected (N = 145) pregnant women at GA ≥ 36 weeks in the Mount Cameroon area

Variable	Category	All n (%)	Infected n (%)
Age group (years)	Less than 21	153 (17.5)	32 (22.0)
	21 to 24	284 (32.5)	51 (35.2)
	Greater than 25	437 (50)	62 (42.8)
Seasonal status	Dry season	397 (45.4)	49 (66.2)
	Rainy season	477 (54.6)	96 (33.8)
Study area status	Semi-rural	441 (50.5)	66 (45.5)
	Semi-urban/urbanized	433 (49.5)	79 (54.5)
Gravidity status	Primigravid	279 (31.9)	63 (43.4)
	Secundigravid	220 (25.2)	41 (28.3)
	Multigravida	375 (42.9)	41 (28.3)
Marital status	Single	271 (31)	45 (31)
	Married	603 (69)	100 (69)
Educational status	At least primary	305 (34.9)	45 (31)
	Secondary	463 (53)	80 (55.2)
	Tertiary	106 (12.1)	20 (13.8)
Occupational status	Business	236 (27)	43 (29.7)
	Civil servant	131 (15)	20 (13.8)
	Farmer	103 (11.8)	15 (10.3)
	Housewife	178 (20.4)	29 (20)
	Student	226 (25.9)	38 (26.2)
ANC status	Less than 4 ANC	321 (36.7)	50 (34.5)
	4 and more ANC	553 (63.3)	95 (65.5)
IPTp-SP status	Zero and one dose	219 (25.1)	39 (26.9)
	Two doses	326 (37.3)	50 (34.5)
	Three and more doses	329 (37.6)	56 (38.6)
ITN status	Yes	696 (79.6)	111 (76.6)
	No	178 (20.4)	34 (23.4)
ITN usage status	Yes	522 (59.7)	83 (57.2)
	No	174 (19.9)	28 (19.3)
	No ITN	178 (20.4)	34 (23.4)
Fever history	Yes	395 (45.2)	63 (43.4)
	No	479 (54.8)	82 (56.6)
Febrile status	Febrile	55 (6.3)	12 (8.3)
	Afebrile	819 (93.7)	133 (91.7)
Anaemic status	Anaemic	509 (58.5)	100 (69)
	Non-anaemic	361 (41.5)	45 (31)

### Prevalence of microscopic and sub-microscopic *Plasmodium falciparum* infection and associated risk factors

Out of the 874 women screened by microscopy for peripheral malaria infection, 67(7.7%) were positive. Among the 67 microscopy-positive cases, the level of parasitaemia ranged from 38 to 162080 parasites/μl giving a geometric mean parasite density of 426/µL. A total of 500 smear negative samples were analysed by PCR. PCR detected 93 (18.6%) women with sub-microscopic *P. falciparum* infection (sub-patent infection). Increased odd of microscopic infection was seen in younger aged women (˂ 21 years) (AOR = 2.89; 95% C 1.29–6.46; P = 0.01) and semi-rural residents (AOR = 2.27; 95% C 1.31–3.96; P = 0.004) compared with older aged and those in the semi-urban/urbanized setting (Table 2). The risk of sub-microscopic parasitaemia increased by three-fold (OR, 3.01; 95% C 1.77 – 5.13; P < 0.001) during the rainy season than in the dry season whereas reduced odds of infection was seen among primigravidae (OR = 0.45; 95% C 0.21–0.94; p = 0.034) and regular ITN users (OR = 0.49; 95% C 0.27–0.90; P = 0.022) compared with multigravidae and non-bed net owners (Table 3). Neither microscopic nor sub-microscopic infection was associated with any dose of SP intake during pregnancy.

**Table 2:** Risk factors of microscopic *P. falciparum* parasitaemia in pregnant women in the mount Cameroon area

Variable	Category	Microscopic infection^#^ [%(n)]		Crude OR (95% CI)	Adjusted OR (95% CI)	P-value
		Positive	Negative			
Age group (years)	< 21	12.4 (19)	87.6 (134)	2.06 (1.11–3.80)	2.89 (1.29–6.46)	0.010
	21–25	7.1 (20)	92.9 (263)	1.10 (0.61–2.00)	1.46 (0.75–2.84)	0.273
	> 25	6.5 (28)	93.5 (406)	REF	REF	
	χ2; p-value	5.903; 0.053				
Seasonal status	Rainy	7.6 (30)	92.4 (367)	0.96 (0.58–1.59)	0.94 (0.56–1.56)	0.797
	Dry	7.8 (37)	92.2 (436)	REF	REF	
	χ2; p-value	0.021; 0.884				
Study setting	Semi-rural	10.7 (47)	89.3 (390)	2.49 (1.45–4.28)	2.27 (1.31–3.96)	0.004
	Semi-urban/urbanized	4.6 (20)	95.4 (413)	REF	REF	
	χ2; p-value	11.256; 0.001				
Gravidity status	Primigravidae	7.9 (22)	92.1 (257)	0.91 (0.52–1.61)	0.56 (0.27–1.18)	0.129
	Secundigravidae	6.0 (13)	94.0 (205)	0.68 (0.35–1.32)	0.55 (0.26–1.15)	0.111
	Multigravidae	8.6 (32)	91.4 (341)	REF	REF	
	χ2; p-value	1.344; 0.511				
IPTp-SP dosage frequency	Zero and one	10.5 (23)	89.5 (196)	1.54 (0.84–2.82)	1.38 (0.74–2.57)	0.318
	Two	6.4 (21)	93.6 (305)	0.90 (0.49–1.67)	0.93 (0.49–1.73)	0.812
	Three and above	7.1 (23)	92.9 (302)	REF	REF	
	χ2; p-value	3.323; 0.190				
ITN usage	Yes	7.7 (40)	92.3 (478)	0.91 (0.49–1.69)	1.15 (0.60–2.18)	0.675
	No	6.9 (12)	93.1 (162)	0.80 (0.37–1.77)	1.07 (0.47–2.40)	0.878
	No ITN	8.4 (15)		91.6 (163)	REF	REF
	χ2; p-value	0.291; 0.865				

IPTp-SP: intermittent preventive treatment in pregnancy with sulfadoxine –pyrimethamine, ITN: insecticide-treated nets, χ^2^ = Person Chi square, OR = Odds Ratio, * Values calculated using confidence interval calculator, ^&^ Values from multinomial regression analysis ^**#**^ Four samples were disqualified

**Table 3:** Risk factors of sub-microscopic *P. falciparum* parasitaemia in pregnant women in the Cameroon area

Variable	Category	Submicroscopic malaria	Crude OR * (95% CI		Adjusted OR ^&^ (95% CI)	P-value
		Positive (%) (n)	Negative (%) (n)			
Age group (years)	< 21	21.9 (16)	78.1 (73)	1.47 (0.77–2.80)	2.02 (0.91–4.77)	0.085
	21–25	21.2 (35)	78.8 (130)	1.41 (0.89–2.32)	1.56 (0.86–2.82)	0.141
	> 25	16.0 (42)	84.0 (220	REF	REF	
	χ2; p-value	2.417; 0.299				
Seasonal status	Rainy	26.0 (70)	74.0 (199)	3.18 (1.91–5.30)	3.01 (1.77–5.13)	< 0.001
	Dry	10.0 (23)	90.0 (208)	REF	REF	
	χ2; p-value	21.186; < 0.001				
Study area	Semi-rural	18.6 (29)	81.4 (127)	0.76 (0.47–1.25)	0.86 (0.51–1.44)	0.564
	Semi-urban	23.0 (64)	77.0 (214)	REF	REF	
	χ2; p-value	18.671 < 0.001				
Gravidity status	Primigravidae	14.2 (22)	85.8 (133)	0.68 (0.39–1.20)	0.45 (0.21–0.94)	0.034
	Secundigravidae	22.3 (29)	77.7 (101)	1.18 (0.69–2.02)	1.01 (0.54–1.88)	0.977
	Multigravidae	19.5 (42)	80.5 (173)	REF	REF	
	χ2; p-value	3.292; 0.193				
Dosage frequency of SP	≤ 1 dose	18.6 (22)	81.6 (147)	1.07 (0.60–1.94)	0.94 (0.50 -1.77)	0.843
	2 doses	19.7 (36)	80.3 (147)	1.15 (0.69–1.92)	1.31 (0.75–2.28)	0.337
	≥ 3 doses	17.6 (35)	82.4 (164)	REF	REF	
	χ2; p-value	0.274; 0.872				
ITN usage	Yes	16.2 (52)	83.8 (269)	0.46 (0.26–0.81)	0.49 (0.27–0.90)	0.022
	No	17.3 (17)	82.7 (81)	0.50 (0.25–1.01)	0.67 (0.31–1.46)	0.312
	No ITN	29.6 (24)	70.4 (57)	REF	REF	
	χ2; p-value	7.832; 0.020				

IPTp-SP: intermittent preventive treatment in pregnancy with sulfadoxine–pyrimethamine, ITN: insecticide-treated nets, χ^2^ = Person Chi square, OR = Odds Ratio, * Values calculated using confidence interval calculator, ^&^ Values from multinomial regression analysis

### Prevalence of *pfdhps* molecular markers of SP resistance

**Fig. 3:**
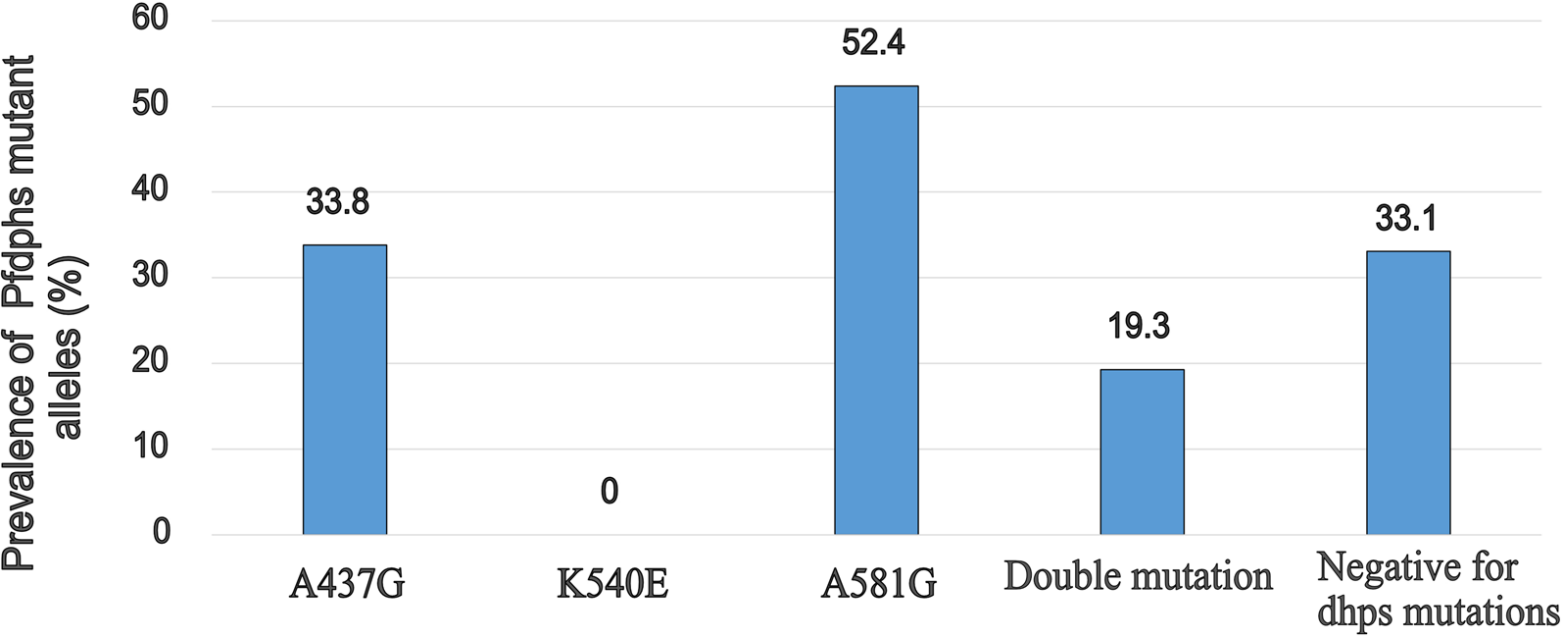
The prevalence of *Pfdhps* mutations at codon 437, 540 and 581 for 145 *P. falciparum* isolates

SNP analysis for *pfdhps* mutations at codon 437, 540 and 581 was successful for 145 DNA samples (52 microscopic and 93 sub-microscopic P*. falciparum* isolates). Overall, many of the isolates haboured the A581G mutation (52.4%; 76) followed by the A437G mutation (33.8%; 49). No isolate had mutation (0%) at codon 540. Amongst the 97 (66.7%) of the *P. falciparum* isolates with at least one mutation, 19.3% (n = 28) had double mutation (A437G + A581G). A total of 48 isolates were negative for the *pfdhps* alleles genotyped (Fig. 3). In general, higher proportion of sub-microscopic (62.4%; 58/93) and microscopic (75%; 39/52) parasite isolates had *pfdhps* mutations associated with resistance to SP.

### Association between IPTp-SP, A437G and A581G gene mutations

**Fig. 4:**
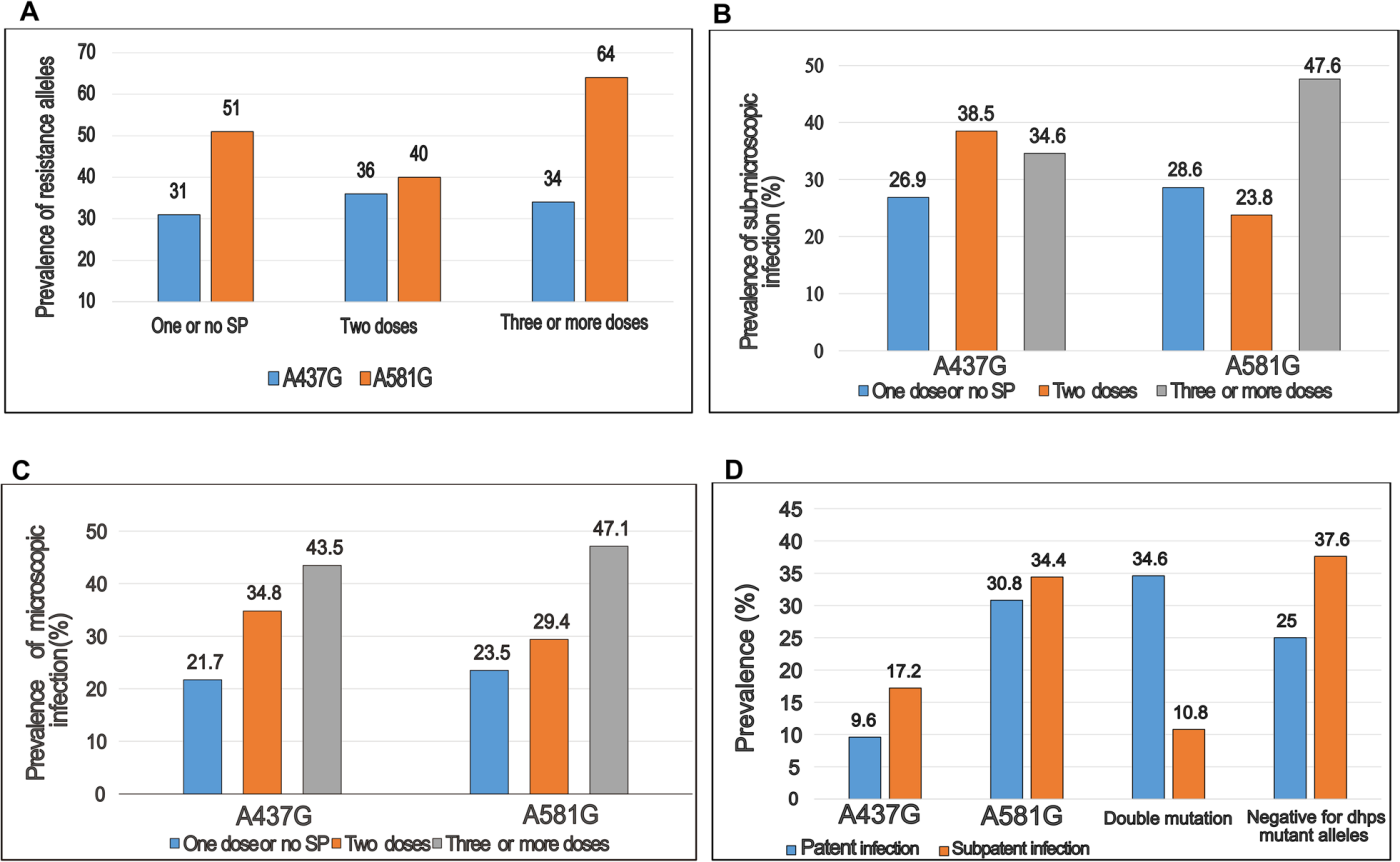
**A** Higher proportion of resistance alleles at DHPS codon 581 is associated with  ≥ 3 doses of SP versus other regimens (p = 0.043); **B** Proportion of A581G sub-microscopic infection is associated with ≥ 3 SP doses (p = 0.027); **C** Proportion of A437G and A581G microscopic infection not different among SP doses (p = 0.874); **D** Double mutation was associated with patent than sub-patent infection outcome (p = 0.005)

The prevalence of parasites carrying the A581G mutation was different between the SP doses compared with those with the A437G mutation. Accordingly, the proportion of A581G was higher in the group that received ≥ 3 (64%) than the 2 (40%) or ≤ 1 (51%) SP dose groups. The difference was significant ((χ ^2^ = 6.27; p = 0.043). After stratifying by malaria parasitaemic status, the positive association between A581G and ≥ 3 doses of SP was evident among the sub-microscopic group (χ ^2^ = 7.2; p = 0.027) (Fig. 4a, b). Also, the relation between A581G mutation and ≥ 3 doses of SP was significant among the multigravidae when compared with primigravidae (χ ^2^ = 11.61; p = 0.003) or paucigravidae) (χ ^2^ = 9.32; p = 0.009) (Additional file 4). For microscopic infection, the proportion of A437G and A581G mutations were similar (χ ^2^ = 0.27; p = 0.874) among SP doses (Fig. 4c). The presence of double mutations was linked with occurrence of microscopic infection (χ ^2^ = 12.9; p = 0.005) when compared with the single mutation of A437G or A581G (Fig. 4d). It worth noting that there was no difference in the baseline characteristics of malaria positive women in the different IPTp-SP groups (Table 4).

**Table 4:** Baseline characteristics of malaria positive women in the different SP groups

Variable	Categories	Dosage frequency of SP [% (n)]			P-value^$^
		≤ 1	2	≥ 3	
Age group (years)	< 21	23.1 (9)	26.0 (13)	17.9 (10)	0.192
	21 – 25	33.3 (13)	44.0 (22)	28.1 (16)	
	> 25	43.6 (17)	30.0 (15)	53.6 (30)	
Season	Rainy	71.8 (28)	66.0 (33)	62.5 (35)	0.641
	Dry	28.2 (11)	34.0 (17)	37.5 (21)	
Study area	Semi-rural	46.2 (18)	40.0 (20)	50.0 (28)	0.585
	Semi-urban	53.8 (21)	60.0 (30)	50.0 (28)	
Gravidity status	Primigravidae	46.2 (18)	36.0 (18)	48.2 (27)	0.479
	Secundigravidae	33.3 (13)	28.0 (14)	25.0 (14)	
	Multigravidae	20.5 (8)	36.0 (18)	26.8 (15)	
ITN usage	Yes	43.6 (17)	52.0 (26)	71.4 (40)	0.052
	No	20.5 (8)	24.0 (12)	14.3 (8)	
	No ITN	35.9 (14)	24.0 (12)	14.3 (8)	

^**$**^ Values from Pearson Chi square test (categorical variables)

### Epidemiological profile, haemoglobin levels, and anaemic status of pregnant women with microscopic versus sub-microscopic *P. falciparum* infection

Infected women with microscopic parasitaemia were more likely (AOR, 7.09; 95% CI 2.59–19.42) from semi-rural setting, experienced a history of fever during pregnancy (AOR, 2.6; 95% CI 1.85–23.96) and to harbour parasites with double mutations (AOR, 6.65; 95% CI 1.07–6.31). Conversely, women with microscopic infection were less likely to have received 2 doses of SP (AOR, 0.29; 95% CI 0.09–0.9) (Table 5). Typically, the microscopic group (9.77 ± 1.49 g/dl) had significantly lower (t = − 2.42; p = 0.017) Hb levels compared with the sub-microscopic group (10.41 ± 6.93 g/dl) regardless of gravidity. A similar pattern was seen for anaemia were a higher prevalence (χ^2^ = 7.13; p = 0.008) occurred in the group with microscopic (82.7%; 43/52) than those with sub-microscopic (61.3%; 57/93) infection. Nevertheless, sub-microscopic parasitaemia (10.41 ± 1.52) in infected women was associated with decreased Hb levels (t = − 2.80; p = 0.005) when compared with those with no infection (10.86 ± 1.37). Similarly, the prevalence of anaemia was higher in the sub-microscopic (61.3%; 57/93) than the non-infected (50.1%; 204/207)) group although the difference was marginal (χ^2^ = 3.78; p = 0.052).

**Table 5:** Epidemiological profile of pregnant women with microscopic parasitaemia versus those with sub-microscopic infection at GA ≥ 36 weeks in the Mount Cameroon area

Variable	Category	Malaria infection status [% (n)]		Unadjusted odds ratio (95% CI)	Adjusted odds ratio (95% CI)	p-value
		Microscopic	Sub-microscopic			
Age group (years)	< 21	50.0 (16)	50.0 (16)	2.10 (0.88–5.03)	1.26 (0.31–5.16)	0.752
	21–25	31.4 (16)	68.6 (35)	0.96 (0.43–2.13)	1.34 (0.44–4.08)	0.603
	> 25	32.3 (20)	67.7 (42)	REF	REF	
Season	Rainy	27.1 (26)	72.9 (70)	0.33 (0.16–0.67)	0.36 (0.14–0.94)	0.036
	Dry	53.1 (26)	46.9 (23)	REF	REF	
Study area	Semi-rural	56.1 (37)	43.9 (29)	5.02 (2.41–10.45)	7.09 (2.59–19.42)	< 0.001
	Semi-urban	19.0 (15)	81.0 (64)	REF	REF	
Gravidity status	Primigravidae	46.3 (19)	53.7 (22)	1.73 (0.77–3.37)	1.13 (0.29–4.37)	0.859
	Secundigravidae	29.3 (12)	70.7 (29)	0.83 (0.35–1.94)	0.67 (0.21–2.13)	0.502
	Multigravidae	33.3 (21)	66.7 (42)	REF	REF	
Dosage frequency of SP	≥ 3 doses	37.5 (21)	62.5 (35)	0.78 (0.34–1.79)	0.49 (0.17–1.41)	0.187
	2 doses	28.0 (14)	72.0 (36)	0.50 (0.21–1.22)	0.29 (0.09–0.90)	0.032
	≤ 1 dose	43.6 (17)	56.4 (22)	REF	REF	
ITN usage	Yes	38.6 (32)	61.4 (51)	1.51 (0.64 – 3.56)	1.81 (0.58–5.66)	0.305
	No	35.7 (10)	64.3 (18)	1.25 (0.38 – 4.08)	4.35 (0.99–19.06	0.052
	No ITN	29.4 (10)	70.6 (24)	REF	REF	
Fever history	Yes	44.4 (28)	55.6 (35)	1.93 (0.97–3.85)	2.60 (1.07–6.31)	0.035
	No	29.3 (24)	70.7 (58)	REF	REF	
DHPS mutation status	A437G only	23.8 (5)	76.2 (16)	0.84 (0.26–2.76)	1.12 (0.28–4.47)	0.875
	A581G only	33.3 (16)	66.7 (32)	1.35 (0.56–3.23)	1.39 (0.48–4.00)	0.546
	Double mutation	64.3 (18)	35.7 (10)	4.85 (1.78–13.19)	6.65 (1.85–23.96)	0.004
	Negative for *dhps* mutation	27.1 (13)	72.9 (35)	REF	REF	

## Discussion

This study explored the effectiveness of IPTp-SP in the Mount Cameroon area in the context of different *P. falciparum* parasitaemic status, *dhps* mutations associated with resistance to SP and malaria transmission intensity. Interestingly, the findings of this study demonstrate the occurrence of sub-microscopic infection revealing its plausible contributory role in malaria transmission and remote SP resistant infection regardless of IPTp-SP dosing. Also, the study reports on the epidemiological profile of women with blood microscopic infection and its negative impact on maternal haemoglobin levels and anaemic status in late pregnancy.

This study revealed that, the prevalence of sub-microscopic *P. falciparum* parasitaemia (18.6%) was more than twice higher than microscopic infection (7.7%) confirming that the parasite burden detected during antenatal clinics is much greater than that predicted by microscopy [16, 17, 19]. The presence of low parasite levels below the microscopic detection threshold may likely reflect an anti-parasitic immune response in these individuals [53]. The development of an efficient parasite-suppressing immune response in multigravidae may explain the significant difference in the prevalence of sub-microscopic parasitaemia between gravidity groups. Compared with multigravidae (≥ 2), primigravidae were less likely to have sub-microscopic infection and accords well with other observations in comparable transmission settings [19, 54]. These submicroscopic infections may reflect prevailing sequestration of parasites in placental intervillous space as shown by some studies [18, 55]. It is also likely that resistance to IPTp-SP may be a major contributing factor to the levels of sub-microscopic carriage in these women as over 60% of the sub-microscopic isolates comprised at least a *dhps* mutation (A437G and/or A581G).

Sub-microscopic malaria has been highly implicated for its contribution to malaria transmission in areas with slide prevalence as low as < 10% or over 20% [56]. The findings showed seasonal change in infection prevalence indicating thrice higher rate of sub-microscopic *P. falciparum* infections in the rainy season than in dry season which conforms other reports [57, 58]. Increased infections during the rainy can be explained by the presence of sufficient vectors [59]. There is evidence demonstrating human-to mosquito transmission in the absence of microscopically detectable parasites [60]. Reduced odds of sub-microscopic parasitaemia was associated with regular ITN usage in concurrence with reports of other studies that revealed higher risk of having infection among non- ITN users [21, 61, 62] indicating proper use of ITN significantly reduces malaria morbidity and mortality [63, 64]. A recent community-based study in the Mount Cameroon area have uncovered a considerable proportion of sub-microscopic malaria infections that go undetected by routine microscopy in children (2–14 years) [62]. Sub-microscopic gametocyte carriers are infectious reservoirs accountable for sustaining *Plasmodium* species between transmission seasons and also may contribute to the stagnation in malaria prevalence [61, 65]. Thus, control programmes in Mount Cameroon region should not relax as sub-microscopic infections persist.

Parasite resistance continues to compromise effectiveness of IPTp-SP throughout sub-Saharan Africa -[6, 7, 9, 66]. Overall, this study did not detect observable protective effect of SP dosing against peripheral microscopic nor sub-microscopic parasitaemia at full term pregnancy. Nevertheless, among the malaria positive cases, lower odds of microscopic parasitaemia was seen in women who received two SP doses compared with those who had ≤ 1 or ≥ 3 doses during pregnancy. In the same area, ≥ 3 SP doses did not ensure optimal anti-plasmodial protection against placental infection [39]. It appears high rates of recrudescent infections occur after the second SP dose, suggesting development of resistance to SP over time [19, 67]. The present study revealed over 50% of the *P. falciparum* isolates with A581G mutation in the Mount Cameroon area where predominance of this highly resistant parasite strain was seen in association with ≥ 3 doses of SP only among sub-microscopic infections and multigravidae in stratified analysis. The association between these mutations and SP dosing is complex [13]. Chang et al. [63] suggested that the use of antimalarial can clear *Plasmodium* infections normally to sub-microscopic levels but persistence of low-level infections following treatment may have implication for the emergence of drug resistant parasites [68]. Also, Harrington et al. [27] suggested a phenomenon of parasite release and facilitation, whereby the most highly resistant parasites out-compete susceptible parasite populations and overgrow under drug pressure in areas with the A581G mutation. Multigravidity may modify development of parasite strains resistant to SP but this remains to be determined. Alternatively, the study data collected may be limited in size to allow for proper interpretation of results. It is worth noting that IPTp-SP continued to protect against LBW [39] in our setting where the prevalence of the A581G mutation is > 50%. Roh et al. [12] suggest that IPTp-SP have potent non-malarial than anti-malarial effects on birthweight. Although trials in eastern Africa reveals DP as a promising alternative drug regimen for IPTp given its long-acting prophylactic effect and highly efficacious antimalarial activity, SP is still superior to DP considering its plausible non-malarial protective effects against poor birth outcomes [12]. Hence, the WHO recommends that IPTp-SP should continue to be used in areas of high SP resistance until more effective alternatives for malaria chemoprevention are found [4].

IPTp-SP fails to inhibit parasite growth where the *Pfdhfr* and *Pfdhps* quintuple mutant acquires an additional *dhps* A581G mutation (a proxy indicator for super-resistant parasites) [69]. In this study, all the isolates with the A581G mutation were found in the absence of the K540E mutation (a proxy for the quintuple *Pfdhfr* and *Pfdhps* mutant) demonstrating that the sextuple mutant conferring a high level of resistance to SP has not fully emerged [33] in the area. Nonetheless, the presence of double mutation with A437G and A581G which was associated with increased odds (6-fold) of microscopic parasitaemia compared to single mutations underscores the role of super-resistant parasites in loss of SP efficacy [26]. The A581G mutation was observed in association with *dhps* A437G and probably other *Pfdhfr* mutations. We did not genotype *Pfdhfr* mutation genes since these are not the focus of WHO policy [3]. More so, these mutations have attained high saturation levels (> 90%) in the mount Cameroon Area [37, 70], The prevalence of K540E remains low (0—2%) in Cameroon [37, 38, 70, 71] and in most West African countries [6, 7, 25, 67]. The implications for the absence of *Pfdhps* K540E mutation in West and some Central African countries remains unknown [6, 7]. Nevertheless, our findings highlight the need for continuous monitoring of the spread of resistance and consideration for research into alternative strategies if A581G mutation spreads.

Increased odds of microscopic infection was observed in individuals living in semi-rural setting. This is in line with studies showing more readily detectable infections where transmission is high. The frequent and super-infection in rural endemic areas may explain increase microscopic parasitaemia in individuals living in these areas [56]. Moreover, new *P. falciparum* clones and increasing clone multiplicities have been associated with clinical episodes, even in individuals with pre-existing asymptomatic infection with other parasite clones [72, 73]. In accord, women with microscopic infection reported a history of febrile illness suggesting infection with new parasite genotypes. The host immune system may be overwhelmed by additional number of different parasite clones and thus elicits only partial cross-immunity against different parasite varieties [74]. The present findings highlight plausible multiplicity of *P. falciparum* infections in pregnant women living in areas of high transmission intensity and thus pose a challenge to control MiP.

Data from this study supports previous works suggesting that sub-microscopic infection affects Hb levels and risk of anaemia [17, 19, 75]. Low-density malaria infection was associated with a decrease in maternal Hb levels. More than half (61.3%) of the participants with sub-microscopic malaria were anaemic, although infection had a marginal effect on anaemia. Anaemia in pregnancy is however multifactorial and can be caused by several factors [76]. Clinical consequences of low-density parasitaemia may be due to direct effects of parasitaemia or to immune dysregulation related to infection. Persistent untreated sub-microscopic infections can allow ensuing pro-inflammatory responses implicated in the pathogenesis of anaemia [77]. Having low-density infection rarely cause febrile illness among individuals including pregnant women in malaria endemic areas and can be attributed to their ability to modulate host inflammation (anti-disease or clinical immunity) as well as capacity to restrict parasite growth (anti-disease immunity) [78, 79]. In line with several studies, the association between microscopic parasitaemia, younger aged group and poor clinical outcomes were expected [19, 34, 43, 80].

### Limitations

This study was limited to the Mount Cameroon area in the Southwest Region of Cameroon and a wider study involving other regions of Cameroon can give a better picture of the evolutionary dynamics of resistance to IPTp-SP at a national level. The skill of the microscopists, quality of the PCR reaction, the amount of genetic material used for PCR can influence the detection of sub-microscopic infections. However, this study involved trained and experienced microscopists and the quality of PCR assay used was sensitive and performed under the supervision of experienced molecular biology scientist. Full *P. falciparum* SP-resistant haplotypes were not genotyped due to logistic reasons and lack of genotyping and sequencing facilities. However, the three mutant alleles genotyped are the focus of WHO policy [3].

## Conclusions

This study showed a higher proportion of sub-microscopic in relation to microscopic *P. falciparum* infection in Mount Cameroon area which suggests assessing parasite carriage with molecular tools is critical in monitoring malaria elimination programmes. The occurrence of sub-microscopic infection is indicative of infection reservoir and potential malaria transmission particularly as mosquitoes can still become infected. ITN use plays a protective role against sub-microscopic parasitaemia, and thus more attention is needed in improving the ITN coverage and usage. The negative impact of sub-microscopic *P. falciparum* infection on maternal Hb levels and anaemia is of particular concern and supports aggressive interventions geared to eliminate this infectious reservoir. The findings did not demonstrate effectiveness of IPTp-SP against peripheral microscopic nor sub-microscopic parasitaemia at delivery The occurrence of resistance mutant (A581G) was associated with SP 3 + doses only among sub-microscopic infections and multigravidae suggesting a complex interaction between parasite mutations and SP dosing. Of public health importance is the contributions of sub-microscopic resistant parasite populations in persistence of malaria infection during pregnancy and the spread of drug resistance in the area. Also, intense malaria transmission may expose women to continues risk of infection and disease. The A540E mutant was absent, it is critical to continue monitoring for the spread of sextuple mutant *P. falciparum* to improve understanding of the influence of this mutation on IPTp-SP effectiveness. As these mutations spread, the protective impact of IPTp-SP will diminish thus the urgent need for therapeutic alternatives to SP, including new drugs or strategies.

## Supplementary Information

**Additional file 1: **Primer sequences for 18s rRNA genes and DHPS mutation genes, amplification conditions and enzymes digest.

**Additional file 2: **Agarose gel documentation of nested PCR 1) for diagnosis of submicroscopic *Plasmodium falciparum* infection. MWM (Molecular weight marker); sample 1 (Positive control); sample 4, 7, 14-17 (submicroscopic *Plasmodium falciparum* positive); sample 2, 3, 5, 6, 8-13, 18, 19 (submicroscopic *Plasmodium falciparum* negative; 2) restriction digestion of nested PCR products for the test of polymorphisms of *Pf*dhps mutant genes: (a) *Ava*II for mutation at codon 437, (b) *Fok*I for mutation at codon 540 and (c) *Bst*UI for mutation at codon 581. MWM: 100bp molecular weight marker.

**Additional file 3: **Characteristics of pregnant women (GA ≥ 36 weeks) living in semi-rural and semi urban/urbanized towns in the Mount Cameroon area.

**Additional file 4: **Association between the A581G mutation and IPTp-SP dosage frequency within gravidity status.

## Data Availability

All datasets generated and analysed during the study are presented in the paper and its additional files.

## Abbreviations

IPTp-SP: Intermittent preventive treatment in pregnancy with sulfadoxine–pyrimethamine
ITN: Insecticide-treated net
dhps: Dihydropteroate synthase
DBS: Dried blood spot
PCR: Polymerase chain reaction
ANC: Antenatal clinic
GA: Gestational age
MMC: Mutengene medical centre
BHC: Bolifamba health centre
MHC: Munyenge integrated health centre
MDH: Muyuka district hospital
