# Supplementary material for: Sub-microscopic Plasmodium falciparum parasitaemia, dihydropteroate synthase (dhps) resistance mutations to sulfadoxine–pyrimethamine, transmission intensity and risk of malaria infection in pregnancy in Mount Cameroon Region
Source: Malar J. 2023 Mar 2;22:73. doi: 10.1186/s12936-023-04485-7 (PMC9979436; doi:10.1186/s12936-023-04485-7)
Supplement: Supplementary file 1 — Additional file 1: Primer sequences for 18s rRNA genes and DHPS mutation genes, amplification conditions and enzymes digest. [file 12936_2023_4485_MOESM1_ESM.docx]

**Additional file 1: Primer sequences for 18s rRNA genes and DHPS mutation genes, amplification conditions and enzymes digest**

| **Gene** | **Mutation** | **Primers** | **Primer sequence** | **PCR conditions** | **Enzyme digest, incubation temperature and time** |
| --- | --- | --- | --- | --- | --- |
| 18s rRNA | Primary | rPlu-5  rPlu-6 | 5' CCTGTTGTTGCCTTAAACTTC 3'  5ʹ TTAAAATTGCAGTTAAAACG 3ʹ | 94°C-3 min, 94°C-30 sec, 55°C-1 min, 68°C-1 min, ×25 cycles, 68°C-3 min, 4°C-hold |  |
|  | Nested | rFal-1  rFal-2 | 5ʹ TTAAACTGGTTTGGGAAAACCAAATATATT 3ʹ  5ʹ ACACAATGAACTCAATCATGACTACCCGTC 3ʹ | 94°C-3 min, 94°C-30 sec, 61°C-1 min, 68°C-1 min, ×30 cycles, 68°C-3 min, 4°C-hold |  |
| DHPS | Primary | R1  R2 | 5' AACCTAAACGTGCTGTTCAA 3'  5' AATTGTGTGATTTGTCCACAA 3' | 94°C-3 min, 94°C-30 sec, 46.5°C-1 min, 72°C-1 min, ×45 cycles, 72°C-3 min, 4°C-hold |  |
|  | Nested A437G | K1  K2 | 5' TGCTAGTGTTATAGATATAGGATGAGCATC 3'  5' CTATAACGAGGTATTGCATTTAATGCAAGAA 3' | 94°C-3 min, 94°C-30 sec, 56.4°C-1 min, 72°C-1 min, ×30 cycles, 72°C-3 min, 4°C-hold | *Ava*II 37°C, 5-15 min |
|  | Nested K540E | K1  K2 | 5' TGCTAGTGTTATAGATATAGGATGAGCATC 3'  5' CTATAACGAGGTATTGCATTTAATGCAAGAA 3' | 94°C-3 min, 94°C-30 sec, 56.4°C-1 min, 72°C-1 min, ×30 cycles, 72°C-3 min, 4°C-hold | *Fok*I, 37°C, 60 min |
|  | Nested A581G | L1  L2 | 5' ATAGGATACTATTTGATATTGGACCAGGATTCG 3'  5' TATTACAACATTTTGATCATTCGCGCAACCGG 3' | 94°C-3 min, 94°C-30 sec, 56.4°C-1 min, 72°C-2 min, ×30, 72°C-3 min, 4°C-hold | *Bst*UI, 60°C, 5-15min |
