# Supplementary figures and images for: Sub-microscopic Plasmodium falciparum parasitaemia, dihydropteroate synthase (dhps) resistance mutations to sulfadoxine–pyrimethamine, transmission intensity and risk of malaria infection in pregnancy in Mount Cameroon Region
Source: Malar J. 2023 Mar 2;22:73. doi: 10.1186/s12936-023-04485-7 (PMC9979436; doi:10.1186/s12936-023-04485-7)

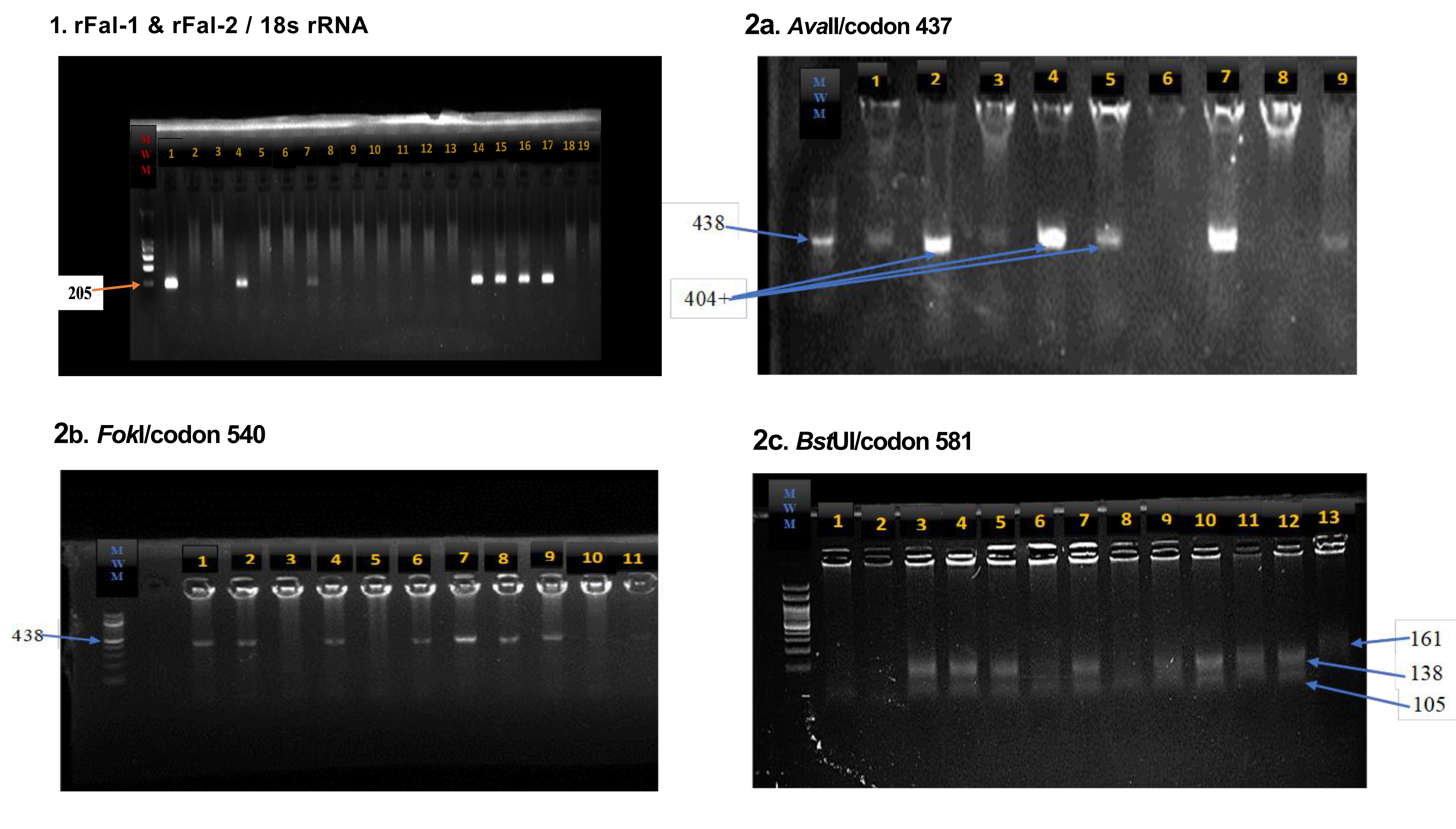

Supplement: Supplementary file 2 — Additional file 2: Agarose gel documentation of nested PCR 1) for diagnosis of submicroscopic Plasmodium falciparum infection. MWM (Molecular weight marker); sample 1 (Positive control); sample 4, 7, 14-17 (submicroscopic Plasmodium falciparum positive); sample 2, 3, 5, 6, 8-13, 18, 19 (submicroscopic Plasmodium falciparum negative; 2) restriction digestion of nested PCR products for the test of polymorphisms of Pfdhps mutant genes: (a) AvaII for mutation at codon 437, (b) FokI for mutation at codon 540 and (c) BstUI for mutation at codon 581. MWM: 100bp molecular weight marker. [file 12936_2023_4485_MOESM2_ESM.jpg]
