# Supplementary material for: Sub-microscopic Plasmodium falciparum parasitaemia, dihydropteroate synthase (dhps) resistance mutations to sulfadoxine–pyrimethamine, transmission intensity and risk of malaria infection in pregnancy in Mount Cameroon Region
Source: Malar J. 2023 Mar 2;22:73. doi: 10.1186/s12936-023-04485-7 (PMC9979436; doi:10.1186/s12936-023-04485-7)
Supplement: Supplementary file 3 — Additional file 3: Characteristics of pregnant women (GA ≥ 36 weeks) living in semi-rural and semi urban/urbanized towns in the Mount Cameroon area. [file 12936_2023_4485_MOESM3_ESM.docx]

**Additional file 3: Characteristics of pregnant women living in semi-rural and semi urbsssssan/urbanised towns in the Mount Cameroon area enrolled at GA ≥ 36 weeks of gestation during ANC clinics**

| **Variable** | Category | **Semi-rural % (n)** | **Semi-urban/urbanised % (n)** | **p-value^$^** |
| --- | --- | --- | --- | --- |
| **Age group (years)** | Less than 21 | 21.1 (93) | 13.9 (60) | 0.019 |
|  | 21 to 24 | 31.3 (138) | 33.7 (146) |  |
|  | Greater than 25 | 47.6 (210) | 52.4 (227) |  |
| **Season** | Dry season | 46.3 (204) | 44.6 (193) | 0.617 |
|  | Rainy season | 53.7 (237) | 55.4 (240) |  |
| **Gravidity status** | Primigravid | 31.7 (140) | 32.1 (139) | 0.850 |
|  | Secundigravid | 24.5 (108) | 25.9 (112) |  |
|  | Multigravida | 43.8 (193) | 42.0 (182) |  |
| **Marital status** | Single | 27.4 (121) | 34.6 (150) | 0.021 |
|  | Married | 72.6 (320) | 65.4 (283) |  |
| **Educational level** | At least primary | 42.6 (188) | 27.0 (117) | < 0.001 |
|  | Secondary | 54.6 (241) | 51.3 (222) |  |
|  | Tertiary | 11.3 (12) | 21.7 (94) |  |
| **Occupation** | Business | 27.2 (120) | 26.8 (116) | < 0.001 |
|  | Civil servant | 2.9 (13) | 27.3 (118) |  |
|  | Farmer | 17.7 (78) | 5.8 (25) |  |
|  | Housewife | 30.2 (133) | 10.4 (45) |  |
|  | Student | 22.0 (97) | 29.8 (129) |  |
| **ANC visits** | Less than 4 ANC | 39.9 (176) | 33.5 (145) | 0.049 |
|  | 4 and more ANC | 60.1 (265) | 66.5 (288) |  |
| **IPTp-SP status** | Zero and one dose | 29.0 (128) | 21.0 (91) | 0.016 |
|  | Two doses | 34.0 (150) | 40.6 (176) |  |
|  | Three and more doses | 37.0 (163) | 38.3 (166) |  |
| **ITN usage** | Yes | 59.0 (260) | 60.5 (262) | < 0.001 |
|  | No | 14.7 (65) | 25.2 (109) |  |
|  | No ITN | 26.3 (116) | 14.3 (62) |  |
| **Fever history** | Yes | 50.6 (223) | 39.7 (172) | 0.001 |
| **Febrile status** | Febrile | 9.5 (42) | 3.0 (13) | < 0.001 |
| **Anaemia status** | Anaemic | 74.1 (324) | 42.7 (185) | < 0.001 |
| **Malaria parasitaemia** | Positive | 10.8 (47) | 4.6 (20) | 0.001 |
| **Parasite density status** | Low | 46.8 (22) | 90.0 (18) | 0.004 |
|  | Moderate | 40.4 (19) | 5.0 (1) |  |
|  | High | 12.8 (6) | 5.0 (1) |  |
| **HIV status** | Positive | 2.7 (12) | 6.5 (28) | 0.008 |

**^$^** Values from Pearson Chi square test (categorical variables)
