# Supplementary material for: Sub-microscopic Plasmodium falciparum parasitaemia, dihydropteroate synthase (dhps) resistance mutations to sulfadoxine–pyrimethamine, transmission intensity and risk of malaria infection in pregnancy in Mount Cameroon Region
Source: Malar J. 2023 Mar 2;22:73. doi: 10.1186/s12936-023-04485-7 (PMC9979436; doi:10.1186/s12936-023-04485-7)
Supplement: Supplementary file 4 — Additional file 4: Association between the A581G mutation and IPTp-SP dosage frequency within gravidity status. [file 12936_2023_4485_MOESM4_ESM.docx]

**Additional file 4: Association between the A581G mutation and IPTp-SP dosage frequency within gravidity status.**

| **Gravidity status** | **A581G**  **mutation status** | **Dosage frequency of SP [% (n)]** | | | | **Gravidity status** | **A581G**  **mutation status** | **Dosage frequency of SP [% (n)]** | | |  |
| --- | --- | --- | --- | --- | --- | --- | --- | --- | --- | --- | --- |
|  |  | **≤ 1** | **2** | **≥ 3** | **χ2**  **p-value** |  |  | **≤ 1** | **2** | **≥ 3** | **χ2**  **p-value** |
| Primigravidae**^$^** | Positive  (23) | 17.4  (4) | 47.8 (11) | 34.8 (8) | 0.351 0.839 | Paucigravidae**^&^** | Positive  (43) | 20.9  (9) | 37.2  (16) | 41.9  (18) | 1.928  0.381 |
|  | Negative  (18) | 22.2  (4) | 38.9  (7) | 38.9  (7) |  |  | Negative  (39) | 30.8  (12) | 41  (16) | 28.2  (11) |  |
| Multigravidae**^#^** | Positive  (41) | 30.2  (16) | 17.0  (8) | 52.8  (28) | **11.611; 0.003** | Multigravidae* | Positive  (33) | 33.3  (11) | 12.1  (4) | 54.5  (18) | **6.274**  **0.009** |
|  | Negative  (51) | 29.4  (15) | 45.1  (23) | 25.5  (13) |  |  | Negative  (30) | 23.3  (7) | 46.7  (14) | 30.9  (9) |  |

$ gravidity = 1; & gravidity ≤ 2; # gravidity ≥ 2; ***** gravidity ≥ 3
